# A Novel VSS-LMS Algorithm Based on Modified Versoria Function for Anti-Jamming

**DOI:** 10.3390/s26031045

**Published:** 2026-02-05

**Authors:** Binghe Tian, Yongxin Feng, Fang Liu, Bixue Song, Sibo Guo

**Affiliations:** Key Laboratory of Information Network and Information Countermeasure Technology of Liaoning Province, Shenyang Ligong University, Shenyang 110159, China; tianbinghe@sylu.edu.cn (B.T.); songbixue@sylu.edu.cn (B.S.); ggsibo@163.com (S.G.)

**Keywords:** adaptive filter, antinoise, VSS-LMS, versoria

## Abstract

In the sensor array signal reception system, improving the accuracy of weak-signal detection is crucial. Traditional fixed-step algorithms struggle to balance the convergence rate (CR) and low steady-state error (SSE) owing to their inherent trade-off limitations. To address this limitation, we propose a novel variable-step-size least-mean-square (VSS-LMS) algorithm based on a modified versoria function, specifically redesigned to enhance curvature characteristics. This approach establishes dynamic coupling between error statistics and step-size factors through nonlinear mapping. It derives closed-loop equations governing parameters (α, β, and γ) relative to the smoothed instantaneous error correlation function. Consequently, an adaptive feedback system is constructed to achieve real-time adjustment through optimal step-size generation. The optimal parameters (α, β, and γ) are determined through empirical enumeration and analysis of their impact on algorithmic performance. Comparative evaluations against established VSS-LMS algorithms confirm performance: the proposed algorithm accelerates convergence while maintaining a low SSE, and exhibits robust signal recovery capabilities under low-SNR conditions with diverse interference types.

## 1. Introduction

The sensor array signal reception system reconstructs base-band information through hybrid signal measurement and computation [1,2,3,4]. This reconstruction process is critically dependent on signal integrity. However, in-band interference within electromagnetic environments (e.g., navigation/communication systems) degrades transmission integrity, causing error accumulation during information recovery [5,6]. As a pivotal front-end filtering technique, adaptive filtering achieves non-ideal signal component suppression through dynamic updates of decision rules [7]. Its principal advantage lies in avoiding strong prior statistical dependencies while dynamically adapting to time-varying channels, thereby ensuring high-fidelity information reconstruction [8]. In addition, adaptive filtering employs delay units and predictor modules to implement interference mitigation techniques, including echo cancellation [9], channel equalization [10,11], system identification [12,13], spectral line enhancement [14,15], and multi-parameter system modeling [16].

Adaptive filtering implementations predominantly leverage algorithms such as LMS [17,18], recursive least squares (RLS) [19,20], and affine projection (AP) [21,22]. Building upon Wiener filtering principles [23], the LMS algorithm introduced by Widrow and Hoff has been widely deployed in adaptive filter systems owing to its computational efficiency, rapid convergence, model-free operation, and superior noise suppression capability. However, as a fixed-step-size algorithm, the LMS inherently exhibits limitations common to conventional fixed-step methods: a fundamental trade-off between the CR and SSE [24]. This performance constraint has motivated the development of variable-step-size (VSS) LMS adaptive filtering algorithms. VSS-LMS adaptive filtering optimizes the performance through real-time step-size adaptation using error signals [25]. This approach employs larger step sizes during the initial high error convergence to accelerate tracking, and then reduces the step sizes near the steady state to minimize residual error.

The design of nonlinear step-size functions commonly employs hyperbolic tangent [26], sigmoid [27,28], and arctangent [29] formulations to balance convergence speed and SSE. To address the limitations of fixed-step-size algorithms, researchers have progressively developed nonlinear-adjusted functions for step-size factor implementations. Huo et al. [30] developed an adaptive filter based on inverse hyperbolic tangent adjustment, balancing CR and SSE but requiring intensive computation due to intrinsic exponential operations. Wang et al. [31] proposed an enhanced adaptive filter utilizing sigmoid-function modification, effectively accelerating CR and thus improving filter performance, but leaving SSE elevated. Wu et al. [32] devised a modified sigmoid VSS-LMS algorithm that enhances CR under large-error conditions while maintaining conventional algorithm accuracy, but demands substantial computational burden. Tong et al. [33] devised an arctangent-based VSS-LMS formulation that stabilizes step-size adaptation in dynamic systems to prevent instability, but incurs substantial computational burden. Guan et al. [34] developed an arctangent-based step-size adjustment method that delivers rapid convergence and tracking under low signal-to-noise ratio (SNR) conditions while maintaining minimal steady misadjustment, but exhibits constrained interference suppression capability.

The versoria function-based VSS-LMS algorithm obviates exponential computations in the step-size factor calculation. By minimizing lookup-table operations, it significantly reduces hardware storage demands, positioning itself as a pivotal advancement in adaptive filtering research. Zhao et al.’s VSS-LMS algorithm [35] integrates variational versoria and Gaussian composite functions. This hybrid approach delivers marked interference suppression and rapid tracking capabilities but incurs elevated computational complexity. Liu et al.’s [36] versoria-based GNSS anti-interference algorithm mitigates the high computational complexity inherent in sigmoid functions while enhancing the receiver interference resistance. Nevertheless, it exhibits compromised tracking capability and suboptimal convergence speeds in both time-varying and time-invariant systems. Consequently, the core innovation of the proposed algorithm lies in the holistic balancing of CR, SSE, computational complexity, and tracking performance, thereby achieving comprehensive system enhancement.

By capitalizing on the computational efficiency of versoria and mitigating its tracking constraints, we formulated a novel VSS-LMS algorithm based on a modified versoria function. The algorithm leverages the computational efficiency of the versoria function to formulate a nonlinear relationship between the error signal and step size variable, thus implementing an efficient VSS adaptive filtering process. The core innovation lies in synergizing the low computational complexity, high implementability, and confined step-size fluctuation characteristics of the versoria function during steady-state operation. This integration enables a novel versoria-like function to adaptively regulate the step-size parameters, establishing a breakthrough in adaptive control mechanisms. The synergistic mechanism achieves a breakthrough performance through two fundamental advances: significantly enhanced robustness via steady-state oscillation suppression, resolving a critical flaw in conventional VSS methods, and accelerated convergence with improved dynamic tracking capability, collectively overcoming the prevailing stability-convergence trade-off.

This paper establishes theoretical and practical advancements by proposing a novel VSS-LMS algorithm based on a modified versoria function. It optimizes critical parameters (α, β, and γ) and validates performance superiority in an operational scenario. The core contributions include:

- Parameter optimization of the proposed algorithm
  - Systematic parameter tuning: By combining step size and weight update, we assess how parameters (α, β, and γ) affect CR and SSE to select optimal values.
  - Analysis of anti-jamming performance: The algorithm exhibits inherent anti-jamming capability due to its immunity to jamming in the autocorrelation term.
- Benchmarking against different algorithms
  - Accelerated convergence: The 40-iteration convergence represents a breakthrough in dynamic system adaptation, where compared algorithms require 2–7× more iterations due to gradient estimation limitations. The 0.026 MSE value further demonstrates robustness against additive white Gaussian noise (AWGN).
  - SSE: Achieves the lowest MSE at both SNR = 5 dB and SNR = 15 dB among six benchmark methods, exhibiting 0.174 and 0.172 improvements respectively over the I-sigmoid algorithm (the highest enhancement observed).
- Anti-jamming performance validation of B1I signals
  - Signal recovery: High-fidelity waveform reconstruction under −24 dB AWGN with a computationally derived 22.76 dB SNR gain, validating robust anti-jamming capability in ultra-low-SNR regimes; achieves 39.75 dB SNR gain at −40 dB input SNR; maintains near-lossless output (20.43 dB at 20 dB input, Δ = 0.43 dB); converges with MSE < 0.1 at 0 dB and 0.01 at 14 dB.
  - Anti-jamming analysis: PSD reduction in single-tone (80.52%), narrowband (75.2%), and sweep jammers (92.13%); 17.12 dB/7.75 dB/5.27 dB gains (versus 21.7 dB/11.3 dB/5.72 dB reference) under single-tone/narrow-band/swept-frequency, confirming the algorithm’s robustness in jamming scenarios.

## 2. Formulation of Adaptive Step-Size Control via Versoria Function

### 2.1. Traditional LMS Adaptive Filter Algorithm

**Figure 1:**
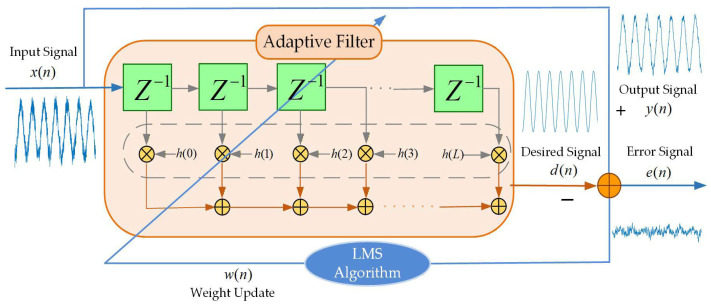
Schematic diagram of LMS adaptive filter.

Figure 1 illustrates the operational principle of the conventional LMS adaptive filter, where the received signal x(n) at iteration n is processed to generate output y(n), with the error signal e(n) defined as the deviation between y(n) and desired signal d(n), and w(n) representing the LMS-adapted weight vector.

For an L-tap adaptive filter, the input signal vector at time n is denoted by X(n)=[x(n), x(n−1),…, x(n−L+1)], with the weight vector of LMS adaptive filter being defined as W(n)=[w(n),w(n−1),…w(n−L+1)]. The output signal y(n) is then computed as the inner product of X(n) and W(n):

(1) $$y(n)=W^{T}(n)X(n)=\sum_{i=1}^{L}w_{i}(n)x_{i}(n)$$

The mean square error (MSE) is a critical performance metric for error signal e(n), given by

(2) $$E\left[e^{2}\left(n\right)\right]=E\left[d^{2}\left(n\right)\right]-2E\left[d\left(n\right)W^{T}\left(n\right)X\left(n\right)\right]+E[W^{T}\left(n\right)X\left(n\right)X^{T}\left(n\right)W\left(n\right)]$$

The update of the weight vector incorporates a gradient factor $\begin{array}{c} \tilde{\nabla } \end{array}$, formalized as

(3) $$\begin{array}{c} W(n+1)=W(n)+\mu (-\tilde{\nabla }(R)) \end{array}$$

where μ denotes the step-size parameter, and

(4) $$\tilde{\nabla }(R)=\frac{\partial (e^{2}(n))}{\partial W(n)}=-2e(n)X(n)$$

denote the gradient estimate of R=[e(n),W(n)].

Substituting (4) into (3), we obtain the weight vector update equation as follows:

(5) W(n+1)=W(n)+2μe(n)X(n)

For guaranteed algorithm convergence, the step-size parameter μ must satisfy

(6) $$0<\mu <\frac{1}{\lambda_{max}}$$

to ensure convergence, where $\lambda_{max}$ denotes the largest eigenvalue of the input autocorrelation matrix.

Table 1 outlines the specific steps of the traditional LMS adaptive filtering algorithm.

### 2.2. Adaptive VSS-LMS Algorithm with Modified Versoria Function

The VSS-LMS algorithm dynamically adjusts the step-size parameter μ(n) based on the instantaneous error signal e(n). This core mechanism effectively resolves the inherent trade-off between CR and SSE in conventional LMS. Specifically, during the initial convergence phase, a large step size (typically approaching a predefined upper bound, $\mu \to \mu_{max}$) is employed to accelerate convergence and enhance tracking capability. As the step size decreases (towards a predefined lower bound, $\mu \to \mu_{min}$), the algorithm approaches to minimize the residual error, thereby achieving low steady misadjustment.

The proposed VSS-LMS algorithm innovates step-size adjustment through three core mechanisms: initially applying a reciprocal transformation to the argument x of the versoria function; subsequently constraining step-size fluctuations within the bounded interval $\left[\mu_{min},\mu_{max}\right]$ using an estimated error autocorrelation (computed from current and prior errors e(n) and e(n−1); and ultimately concurrently suppressing noise while guaranteeing convergence and minimizing steady misadjustment.

**Figure 2:**
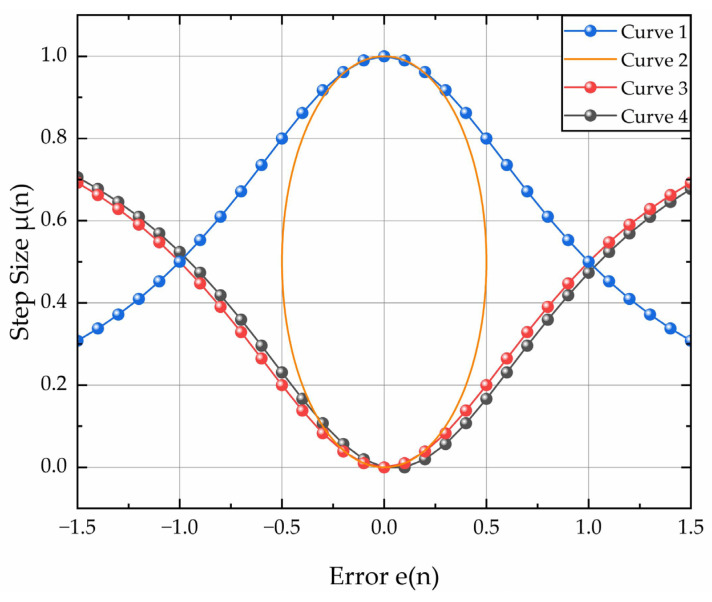
Adjustment procedure of standard versoria function.

Figure 2 illustrates the adjustment procedure for the standard versoria function. The baseline function (Curve 1), defined by $f(x)=\frac{1}{1+x^{2}}$, undergoes a geometric transformation via a tangential circle with radius r=0.5, yielding Curve 2 with the expression $x^{2}+(f(x)-\frac{1}{2}{)}^{2}=\frac{1}{4}$. Subsequent parameter e(n) discretization with value Δe(n)=0.1 generates two derived curves: Curve 3, obtained by applying reciprocal mapping to the independent variable ($x\to x^{-1}$), and Curve 4, incorporating autocorrelation-based step factor modification. The mathematical representation of Curve 4 is given by $\mu (n)=\frac{1}{1+\frac{1}{E(e(n)e(n-1))}}$.

Figure 2 experimentally validates the compliance of Curve 4 with dynamic step-size adjustment specifications for real-time sensor signal processing. The control coefficients α, β, and γ in (7) govern the convergence trajectory and SSE, which are critical for sensor applications in dynamic noise environments.

(7) $$\mu (n)=\alpha \left(\frac{1}{1+\frac{1}{\beta E[e(n)e(n-1){]}^{\gamma }}}\right)$$

where α > 0, β > 0, and γ > 0, parameters α and β jointly determine the domain of μ(n), while coefficient γ governs its gradient characteristics.

Given the constraints in (7), the iterative algorithm is constructed by (8)–(10).

(8) $$e(n)=d(n)-X^{T}(n)W(n)$$

(9) $$\mu (n)=\alpha \left(\frac{1}{1+\frac{1}{\beta E[e(n)e(n-1){]}^{\gamma }}}\right)$$

(10) w(n+1)=w(n)+2μe(n)X(n)

## 3. Parameter Optimization and Anti-Jamming Performance Analysis of Proposed Algorithm

The proposed algorithm dynamically adjusts the step-size bounds $\left[\mu_{min},\mu_{max}\right]$ through parameters α and β, while the coefficient γ tunes the convergence speed, enabling real-time adaptive step-size control.

### 3.1. Performance-Oriented Parameter Tuning

To determine optimal values and functional forms of parameters α, β, and γ in the proposed step-size function, we quantify their impacts on CR, SSE, and steady misadjustment through rigorous analysis of step-size dynamics and weight update trajectories.

Simulation conditions are configured as follows:

- Adaptive filter order: 2;
- Initial weight vector: $W_{0}=[0.8,\;0.5{]}^{T}$;
- At iteration 500: weight reset to $W_{1}=[0.4,\;0.2{]}^{T}$ for steady misadjustment analysis in a dynamic system;
- Input signal: X(n)∼N(0,1);
- Interference noise: $I(n)∼N(0,\;10^{-3})$;
- Statistical property: X(n) and I(n) are statistically uncorrelated;
- Monte Carlo trials: 200 independent runs with 1000 iterations each.

**Figure 3:**
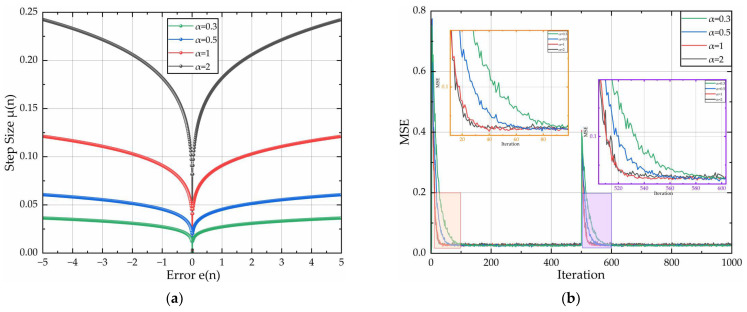
Relationship curves for μ(n) and e(n) & convergence speed curves of the algorithm: (**a**) adjustment curves of μ(n) and e(n); (**b**) algorithm convergence speed diagram for different α.

With parameters β = 0.1 and γ = 0.1 held constant, Figure 3 quantifies the curves of step-size factor μ(n) versus instantaneous error e(n), and convergence speed curves with discrete α∈{0.2, 0.5, 1,2}. Figure 3a indicates that parameter α significantly governs the adaptation dynamics of μ(n). For a fixed e(n), larger values of parameter α accelerate transient convergence but induce pronounced step-size discontinuities that cause dynamic destabilization. Consequently, excessively large values of α should be avoided to prevent dynamic destabilization. As demonstrated in [19], steady misadjustment exhibits a strong positive correlation with step size. Reducing the magnitude of parameter α produces a proportional reduction in steady misadjustment. However, sub-threshold parameter values trigger drastic convergence deceleration, while diminished step size concurrently elevates both SSE and steady misadjustment. As evidenced in Figure 3b, convergence accelerates with increasing α at fixed MSE levels. Owing to the diminishing bounding efficiency, the convergence improvement becomes insignificant beyond α = 1. Thus, optimal design efficiency is achieved at α = 1.

**Figure 4:**
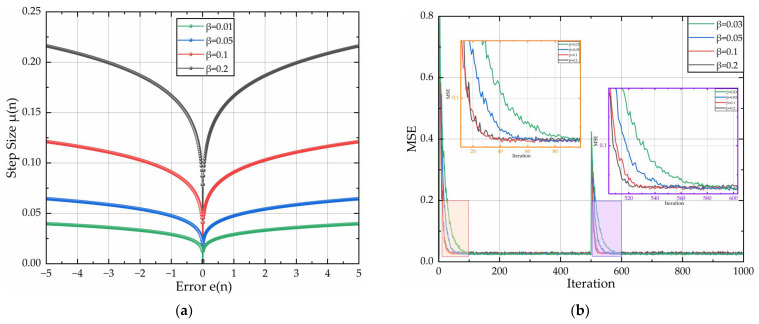
Relationship curves for μ(n) and e(n) & convergence speed curves of the algorithm: (**a**) adjustment curves of μ(n) and e(n); (**b**) algorithm convergence speed diagram for different β.

With parameters α = 1 and γ = 0.1 held constant, Figure 4 quantifies the curves of step-size factor μ(n) versus current error e(n), and convergence speed curves with discrete β∈{0.01, 0.05, 0.1, 0.2}. Figure 4a reveals that parameter β exhibits control characteristics analogous to parameter α. Therefore, parameter β should avoid excessively large values, while sub-threshold β values trigger convergence deceleration. Critically, diminished step size elevates steady misadjustment. As evidenced in Figure 4b, convergence accelerates with increasing β at a fixed MSE level. Convergence gains saturate for β > 0.1 with marginal improvement. Therefore, the optimal design efficiency is achieved β = 0.1.

**Figure 5:**
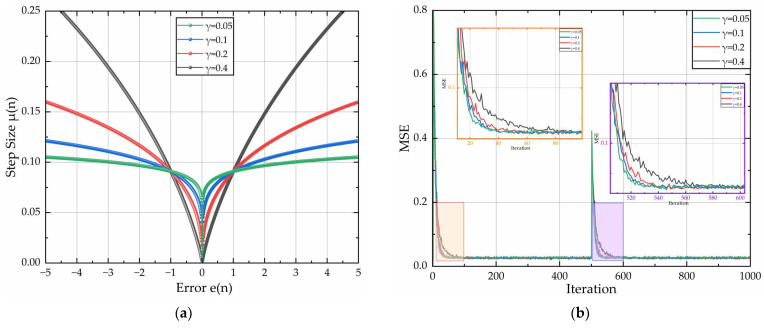
Relationship curves for μ(n) and e(n) & convergence speed curves of the algorithm: (**a**) adjustment curves of μ(n) and e(n); (**b**) algorithm convergence speed diagram for different γ.

With parameters α = 1 and β = 0.1 held constant, Figure 5 quantifies the curves of step-size factor μ(n) versus current error e(n), and convergence speed curves with discrete γ∈{0.05,0.1,0.2,0.4}. Figure 5a indicates that the parameter significantly governs the shape of μ(n). Smaller values of parameter γ increase the slope of the curve, and accelerate the convergence rate for a fixed e(n). As e(n)→0, the increments of step size diminish. However, excessively large γ values induce pronounced step-size discontinuities that cause dynamic destabilization. Thus, an excessively small value of parameter γ must be avoided. Nevertheless, the oversized value of parameter γ triggers drastic convergence deceleration, while contracted step-size intervals elevate steady misadjustment. Figure 5b demonstrates convergence acceleration with decreasing γ at fixed MSE levels, but improvements saturate for γ < 0.1. Therefore, the optimal design efficiency is achieved γ = 0.1.

### 3.2. Algorithm Analysis of Anti-Jamming Performance

As derived in Equation (8), the desired signal d(n) is mathematically represented by Equation (11).

(11) $$d(n)=e(n)+X^{T}(n)W(n)$$

The desired signal d(n) is decomposed as the sum of the jamming signal and compensation signal:

(12) $$d(n)=I(n)+X^{T}(n)W_{\xi }(n)$$

where $I(n)∼N(0,\sigma^{2})$ denotes the zero-mean Gaussian white noise statistically independent of the input signal X(n), $W_{\xi }(n)$ represents the optimal weight vector at the discrete-time index n.

Subtracting (12) from (11) yields

(13) $$e(n)=I(n)+X^{T}(n)(W_{\xi }(n)-W(n)$$

Defining the weight increment $\Delta W(n)=W_{\xi }(n)-W(n)$, the error term e(n) is

(14) $$e(n)=I(n)+X^{T}(n)\Delta W(n)$$

Consequently, parameter e(n−1) admits the representation:

(15) $$e(n-1)=I(n-1)+X^{T}(n-1)\Delta W(n)$$

Furthermore,

(16) $$\begin{array}{l} e(n)e(n-1)\\ =(I(n)+X^{T}(n)\Delta W(n))(I(n-1)+X^{T}(n-1)\Delta W(n-1))\\ =I(n)I(n-1)+I(n)X^{T}(n-1)\Delta W(n-1)+\\ \;I(n-1)X^{T}(n)\Delta W(n)+X^{T}(n)\Delta W(n)X^{T}(n-1)\Delta W(n-1) \end{array}$$

Since I(n) exhibits zero mean and is independent of X(n), taking expectation on both sides yields the result formalized in Equation (17).

(17) $$E[e(n)e(n-1)]=E[X^{T}(n)\Delta W(n)X^{T}(n-1)\Delta W(n-1)]$$

This derivation demonstrates that E[e(n)e(n−1)] remains statistically immune to I(n), confirming inherent anti-jamming capability.

## 4. Experimental Simulation and Analysis

To comprehensively evaluate the performance of the proposed algorithm, MATLAB R2020a simulations were conducted in three phases. The anti-jamming effectiveness of the algorithm was quantified as follows:

- CR/SSE analysis: The CR and SSE of the proposed algorithm were benchmarked against existing VSS-LMS methods [30,31,32,33,34] by analyzing MSE as a function of iteration index. This comparative analysis explicitly evaluates the performance of algorithms in dynamic systems.
- Anti-noise robustness: The average MSE value served as the metric to quantify noise resilience of the proposed method under varying SNRs (SNR = 5 dB, 15 dB).
- Navigation scenario validation: A dedicated simulation platform generated B1I signals, with signal recovery fidelity evaluated across domains:
  - AWGN-corrupted signal analysis: Time-domain waveform distortion suppression quantification and algorithm performance characterization across varying SNR regimes.
  - Jamming (single-tone, narrow-band, and swept-frequency) signal analysis: power spectral density (PSD)-based jamming resistance validation, time frequency (TF) suppression efficacy against interference, and temporal signal-to-interference ratio(SIR) enhancement evolution analysis under interference scenarios.

Reproducible parameter configurations for all the VSS-LMS algorithms are provided in Table 2.

### 4.1. Performance Analysis of VSS-LMS Algorithms in Dynamic Systems

To assess the algorithm’s influence on convergence speed, SSE, and tracking capability in a dynamic system, simulations were executed across both time-varying and time-invariant systems. All the experiments adhered to the experimental configuration described in Section 3.2.

**Figure 6:**
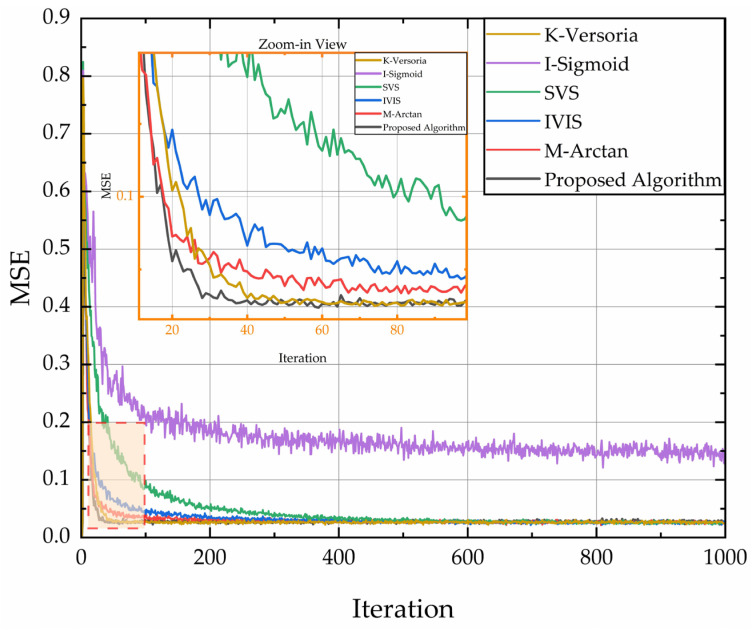
Comparison of MSE learning curves of different algorithms in a time-invariant system.

The filter transfer function was configured with second-order poles, initialized with a weight vector $W_{0}=[0.8,\;0.5{]}^{T}$. The input signal X(n) was modeled as standard white Gaussian noise N(0,1). Environmental interference noise I(n)∼N(0,0.01). Both vectors are statistically independent. Each simulation was executed 1000 iterations with 200 Monte Carlo trials per curve. For the time-varying system, an abrupt change in adaptive weights to $W_{1}=[0.4,\;0.2{]}^{T}$ was triggered at iteration 500. Comparative MSE learning curves under these distinct dynamic regimes are presented in Figure 6.

Figure 6 compares the MSE learning curves of different algorithms in time-invariant systems. After multiple iterations, the steady-state MSE of I-Sigmoid stabilizes at 0.15, while others (K-Versoria/SVS/IVIS/M-arctan) match our method’s 0.026 MSE. Regarding initial convergence speed, convergence required only 40 iterations for our method versus 50 (K-Versoria), 240 (I-Sigmoid), 300 (SVS), 100 (IVIS), and 75 (M-arctan). These results demonstrate faster convergence with a lower SSE.

**Figure 7:**
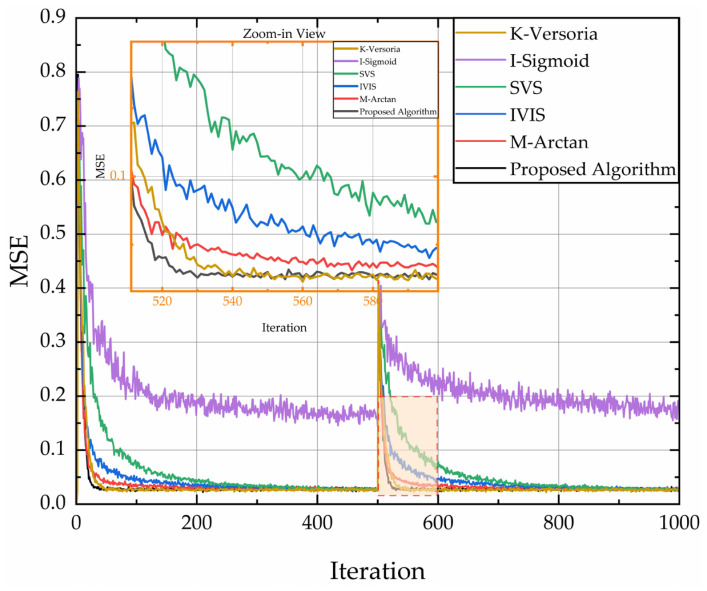
Comparison of MSE learning curves of different algorithms in time-varing system.

Figure 7 compares MSE learning curves in time-varying systems. After the abrupt system change at iteration 500, I-Sigmoid exhibits the highest SSE and weakest tracking capability. In contrast, the proposed algorithm achieves minimal SSE and demonstrates superior tracking performance.

### 4.2. Anti-Noise Performance Evaluation of VSS-LMS Algorithms

**Figure 8:**
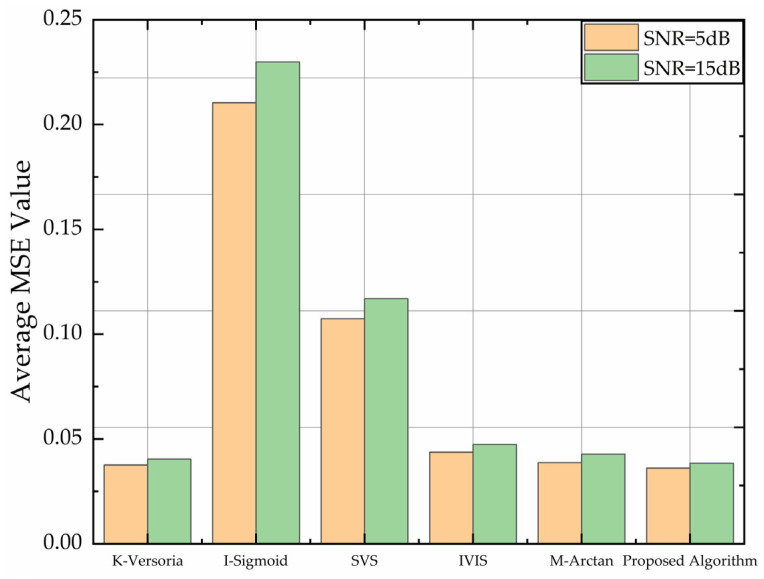
Comparison of anti-noise ability of different algorithms.

At convergence, a lower MSE indicates stronger resilience to jamming. To evaluate the anti-jamming performance of our algorithm, we injected additive background noise into the received signals at SNRs of −5 dB and 15 dB. All the other parameter settings remained consistent with those specified in Section 3.2. The average MSE values after convergence were used as the primary performance metric, and the comparative results are presented in Figure 8.

As observed in Figure 8, increasing SNR reduces average MSE values, enhancing jamming resilience across all the algorithms, consistent with communication theory. The I-Sigmoid algorithm exhibits relatively poor performance due to its slow convergence, leading to higher MSE values. Conversely, the statistical independence between input signal X(n) and noise I(n) enables our algorithm to achieve minimal MSE at both SNR = 5 dB and SNR = 15 dB. These results demonstrate superior robustness against noise.

### 4.3. BeiDou Navigation Scenario: Multi-Domain Assessment of B1I Signal Restoration Capability

To further evaluate anti-jamming performance, the received sample signal is modeled as

(18) $$Sig_{BD}(n)=AC(n)D(n)\cos(2\pi f_{1}n+\varphi )$$

where

- ${Sig}_{BD}(n)$: B1I signal;
- A: Amplitude factor;
- C(n): Pseudo-random code;
- D(n): Data modulation code;
- $f_{1}$: Center frequency;
- φ: Initial carrier phase.

Case 1: The input signal X(n) represents the $Sig_{BD}(n)$. Background noise I(n) is modeled as AWGN with $I(n)\sim N(0,\sigma^{2})$, where the variance $\sigma^{2}$ is calibrated to achieve an SNR of −24 dB over the entire sequence, sampling frequency is set at 100 MHZ. The SNR is defined as

(19) $${SNR}_{BD}=10{log}_{10}(\frac{P_{sig}}{P_{noise}})$$

with $P_{sig}$ denoting the average power of X(n), and $P_{noise}$ = $\sigma^{2}$ for AWGN.

**Figure 9:**
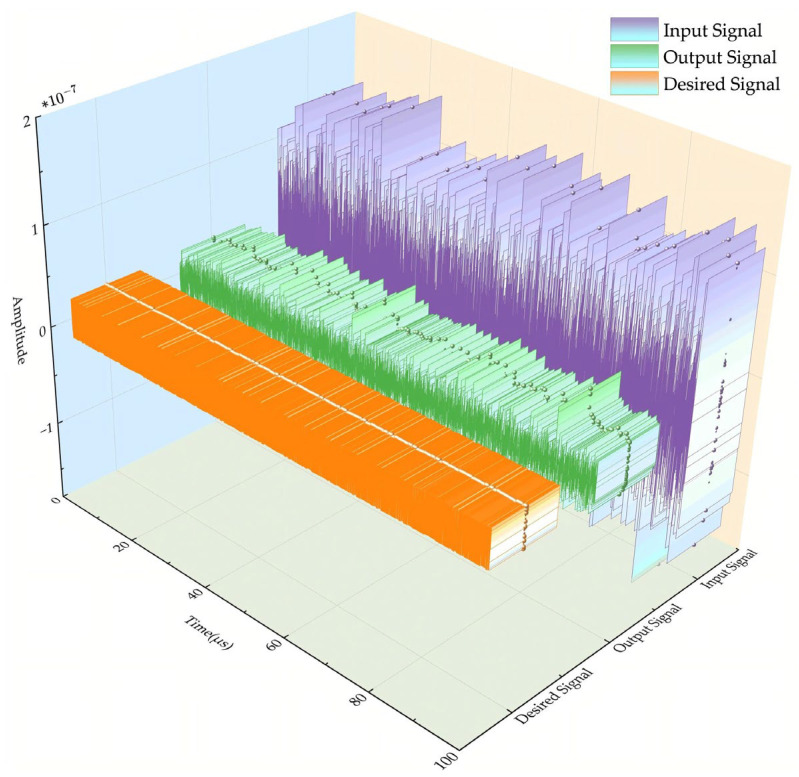
Signal time-domain comparison waveform with 10,000-point AWGN.

Figure 9 shows the time-domain waveform (10,000 points) comparison of the BeiDou B1I signal under AWGN. The proposed algorithm enables order-of-magnitude attenuation of the input signal’s time-domain amplitude. Consequently, the attenuation yields high-fidelity output amplitude conforming to the desired signal profile under AWGN.

**Figure 10:**
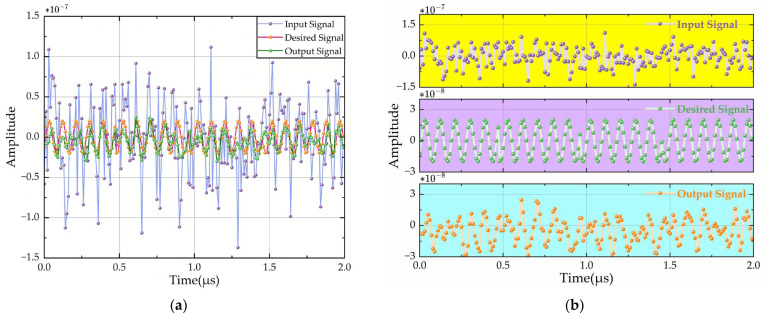
Signal time-domain comparison waveform with 200-point AWGN: (**a**) superimposed waveforms; (**b**) isolated waveforms.

For localized analysis, Figure 10 compares the initial 200 samples of the signal sequence: Figure 10a presents time-domain waveforms of the input, desired, and output signals, while Figure 10b displays their corresponding isolated waveform components. The computationally derived 22.76 dB SNR gain validates robust anti-interference performance under low-SNR conditions.

**Figure 11:**
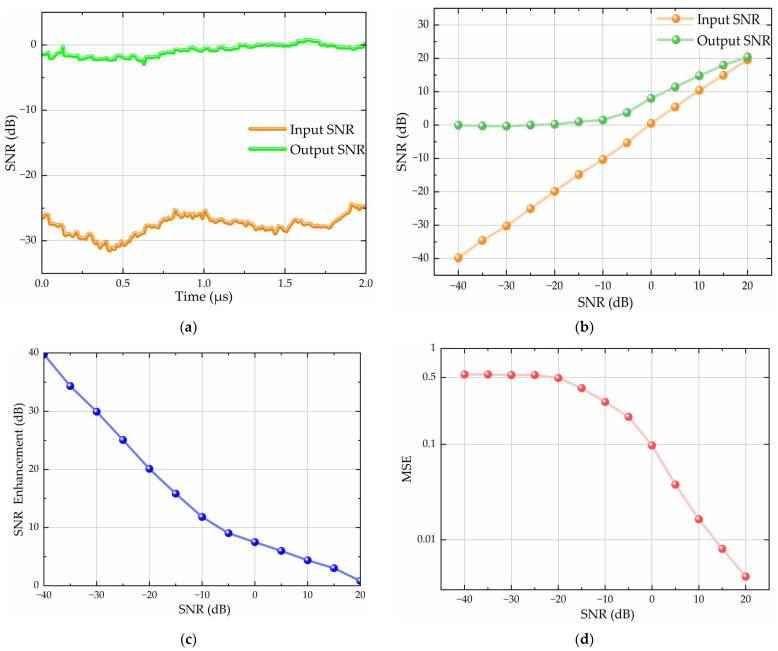
Performance evaluation of the proposed algorithm under varying SNR conditions (−40 dB to 20 dB): (**a**) SNR trajectory at 30 dB of input vs. output SNR; (**b**) input vs. output SNR comparison; (**c**) SNR enhancement curve; (**d**) MSE evolution curve.

Figure 11 shows the performance of the proposed algorithm under varying SNR conditions (−40 dB to 20 dB). These results demonstrate significant performance improvements across this range. Figure 11a characterizes the input SNR trajectory at 30 dB of input vs. output SNR, revealing a 30.18 dB enhancement that validates the algorithm’s anti-interference performance in low-SNR regimes. Figure 11b,c reveal a robust signal recovery capability across a dynamic SNR range. In the low-SNR regime (−40 dB to −10 dB), the output SNR exhibited nonlinear stabilization with 39.75 dB enhancement at −40 dB (input = −40 dB vs. output = −0.25 dB), maintaining >11.61 dB enhancement at −10 dB (input = −10 dB vs. output = 1.61 dB). In the high-SNR transition regime (−10 dB to 20 dB), the enhancement gradually declined from 11.61 dB at −10 dB to 0.43 dB at 20 dB (input = 20 dB vs. output =20.43 dB), while the output SNR asymptotically approached the input SNR, validating near-lossless recovery. Figure 11d shows the MSE evolution curve. The results reveal that the proposed algorithm achieves MSE stability under ultra-low SNR conditions (−40 to −20 dB). Its adaptive precision enhancement drives MSE to <0.1 at 0 dB SNR and converges to 0.01 at 14 dB. The full-range adaptability from noise-dominated to clean-signal regimes establishes new benchmarks for robust signal recovery.

Case 2: The input signal X(n) denotes the $Sig_{BD}(n)$. The interference signal J(n) comprises three types of interference, as defined in Table 3. J(n) is applied at a jammer-to-signal ratio (JSR) of 10 dB applied across the $Sig_{BD}(n)$ sequence. The sampling frequency is fixed at $f_{s}$ = 16.368 MHZ. Mathematical formulations and parameter distributions for each interference type are specified in Table 3.

**Figure 12:**
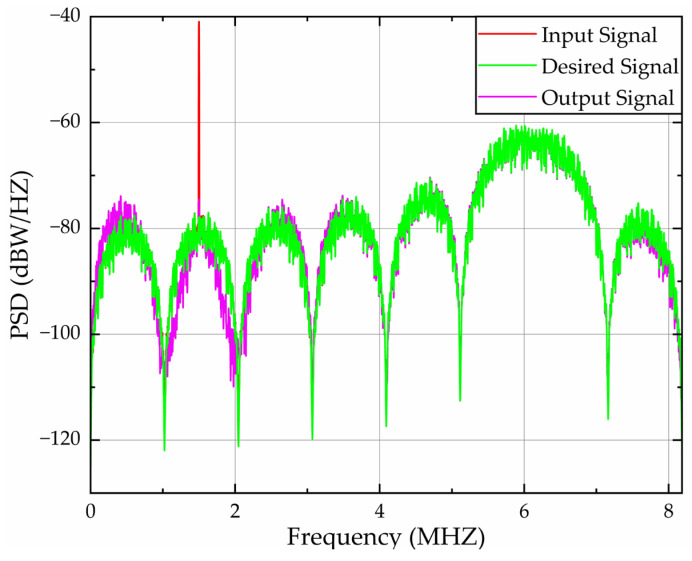
PSD validation of filtering algorithms under single-tone interference.

Figure 12 quantifies spectral processing efficacy through PSD analysis across the 0–8.184 MHz bandwidth under single-tone interference centered at 1.5 MHz. The results demonstrate exceptional spectral congruence between input and desired signals, as evidenced by near-identical peak alignment. Crucially, the algorithm achieves:

- A 33.56 dBW/Hz peak suppression at 1.5 MHz, reducing input PSD from −40.93 dBW/Hz to −74.49 dBW/Hz at the output;
- The error PSD is constrained to 8.11 dBW/Hz at 1.5 MHz, representing an 80.52% amplitude reduction relative to the initial error between the input and desired PSD (−41.67 dBW/Hz).

**Figure 13:**
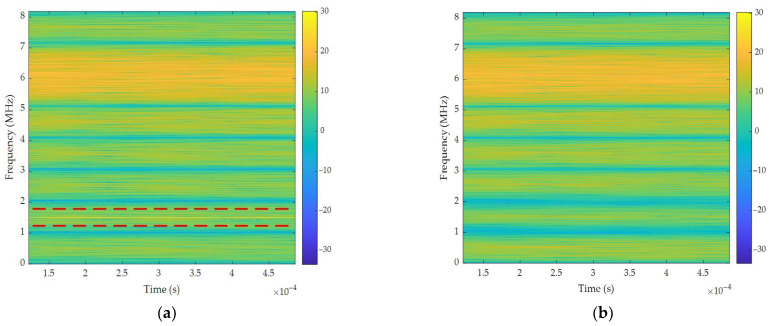
TF analysis of single-tone interference suppression: (**a**) input signal; (**b**) output signal.

Figure 13 presents the TF analysis of single-tone interference suppression centered at 1.5 MHz. This visualization provides compelling evidence of the algorithm’s interference suppression capability. As shown in Figure 13a, a prominent single-tone interference manifests as a high-energy component at 1.5 MHz within a 3.63 ms duration. Crucially, single-tone interference obscured the desired signal components concentrated at 1.5 kHz. Figure 13b shows the output TF distribution processed by the proposed filtering algorithm. Single-tone interference is visually negligible in this representation, demonstrating significant suppression across the frequency band.

**Figure 14:**
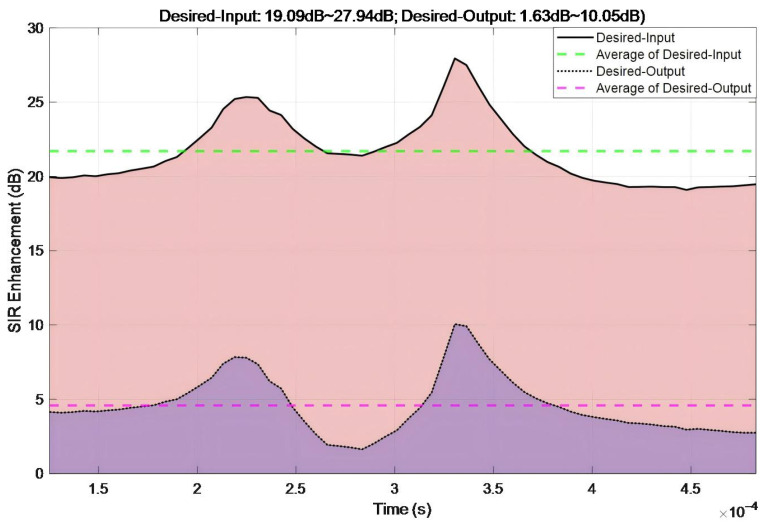
Temporal evolution of SNR enhancement under single-tone interference.

Figure 14 illustrates the temporal evolution of SIR enhancement under single-tone interference. The Desired-Input SIR values ranged from 19.09 to 27.94 dB with a mean value of 21.7 dB. The Desired-Output SIR values fall within the range of 1.63–10.05 dB with a mean value of 4.58 dB. The proposed algorithm induces SIR reduction (output–input) fluctuates between 15.8 and 19.77 dB across measured points, with an average reduction of 17.12 dB. These metrics conclusively validate the efficacy of the proposed algorithm for single-tone interference.

**Figure 15:**
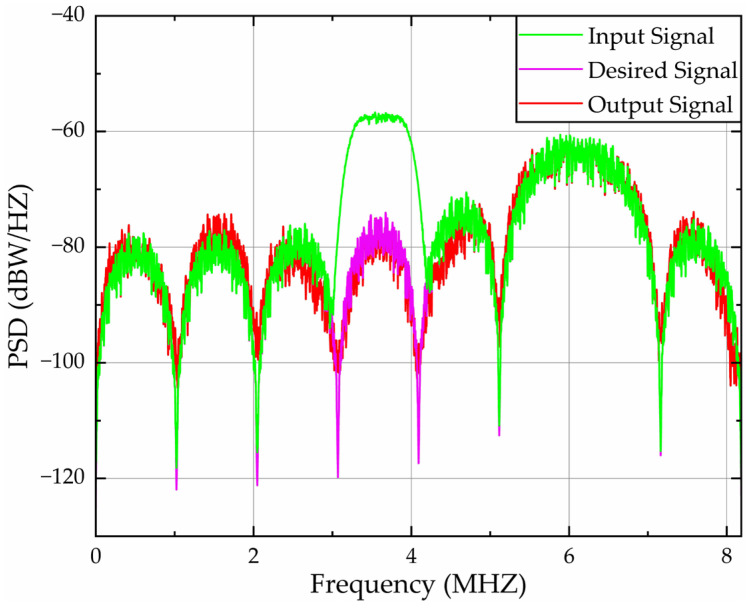
PSD validation of filtering algorithms under narrow-band interference.

Figure 15 depicts PSD analysis over 0–8.184 MHz under narrow-band interference across a 200 kHz band centered at 1.2 MHz (1.1–1.3 MHz). Table 4 lists the quantitative PSD characteristics of narrow-band interference. Joint analysis reveals that the algorithm achieves:

- A 16.76 dB average peak suppression within the 1.1–1.3 MHz band, reducing the average input PSD from −65.40 dBW/Hz to −82.16 dBW/Hz at the output;
- Constrained average error PSD to 5.53 dBW/Hz over 1.1–1.3 MHz, corresponding to a 75.2% reduction in amplitude relative to the initial error between the input and desired PSD.

**Figure 16:**
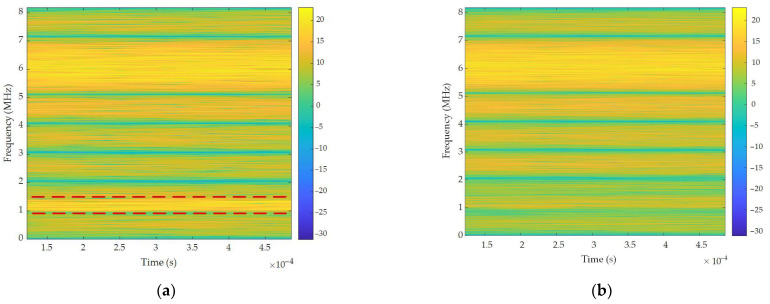
TF analysis of narrow-band interference suppression: (**a**) input signal; (**b**) output signal.

Figure 16 presents the TF analysis of narrow-band interference suppression within the 1.1–1.3 MHz band. As evidenced in Figure 16a, persistent narrow-band interference manifests as a continuous high-energy distribution spanning this frequency range. Narrow-band interference critically obscures desired signal components centered at 1.2 MHz. Following algorithmic processing, Figure 16b demonstrates the visual elimination of interference energy across the 1.1–1.3 MHz band. Concurrently, previously obscured signal features were successfully recovered. These results collectively confirm the spectral containment capability of the proposed algorithm for narrow-band interference mitigation.

**Figure 17:**
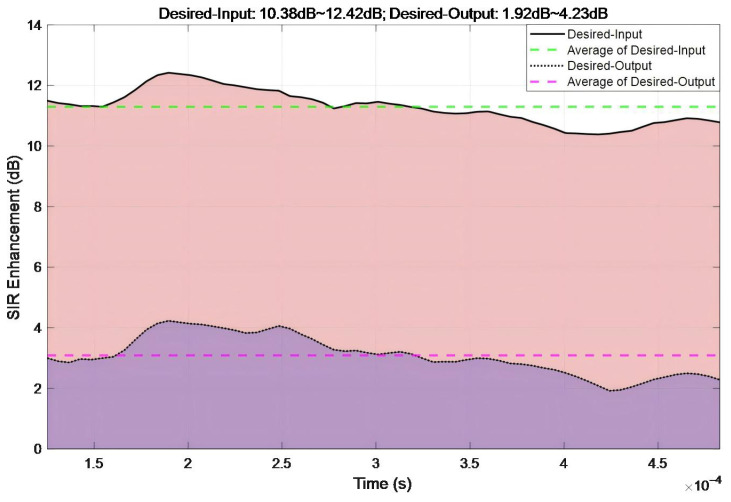
Temporal evolution of SNR enhancement under narrow-band interference.

Figure 17 depicts the temporal evolution of SIR enhancement under narrow-band interference. The Desired-Input SIR ranged from 10.38 to 12.42 dB, with a mean value of 11.3 dB. In contrast, the Desired-Output SIR varied between 1.92 and 4.23 dB, yielding a mean of 3.04 dB. The SIR reduction induced by the proposed algorithm (output–input) fluctuates from 6.92 to 8.89 dB across the measured points, averaging 7.75 dB. These results demonstrate the efficacy of the proposed algorithm in mitigating narrow-band interference.

**Figure 18:**
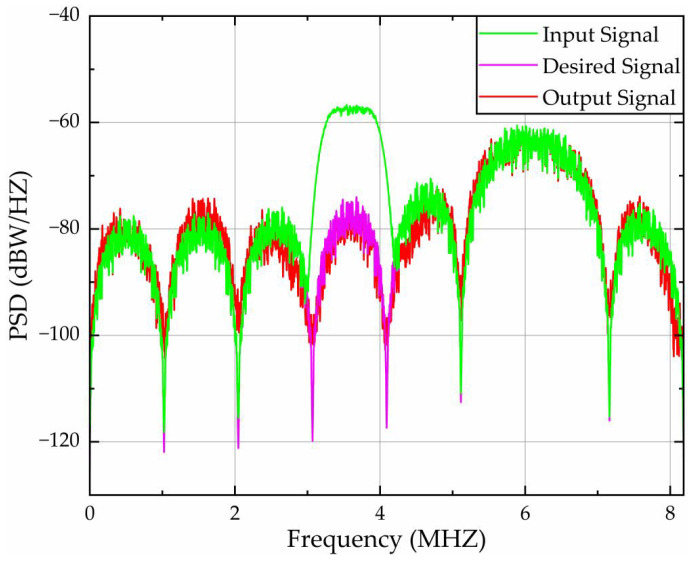
PSD validation of filtering algorithms under frequency-swept interference.

Figure 18 presents PSD analysis over 0–8.184 MHz under frequency-swept interference targeting the 3–4 MHz band. With quantitative metrics detailed in Table 5, the proposed algorithm achieves:

- A 23.2 dB average peak suppression within the interference band 3–4 MHz, reducing mean PSD from −61.6 dBW/Hz (input) to −84.8 dBW/Hz (output);
- A 0.45 dBW/Hz average error PSD over 3–4 MHz, corresponding to a 92.13% amplitude error reduction relative to the initial input–desired PSD discrepancy.

**Figure 19:**
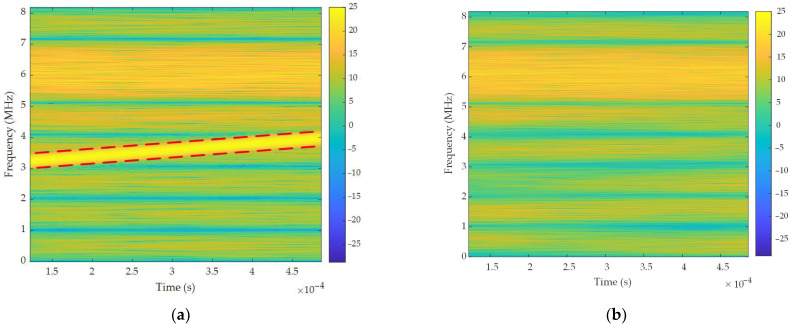
TF analysis of frequency-swept interference suppression: (**a**) input signal; (**b**) output signal.

Figure 19 evaluates TF suppression efficacy against frequency-swept interference in the 3–4 MHz band. Figure 19a reveals a characteristic linear sweep signature, manifested as a high-energy, time-evolving ridge traversing the band that severely masks underlying signals. Post-processing in Figure 19b effectively eradicates this sweep trajectory by eliminating the time-varying ridge and restoring the spectral integrity within the band. This contrast validates the capacity of the algorithm to isolate dynamically swept interference while preserving signal fidelity.

**Figure 20:**
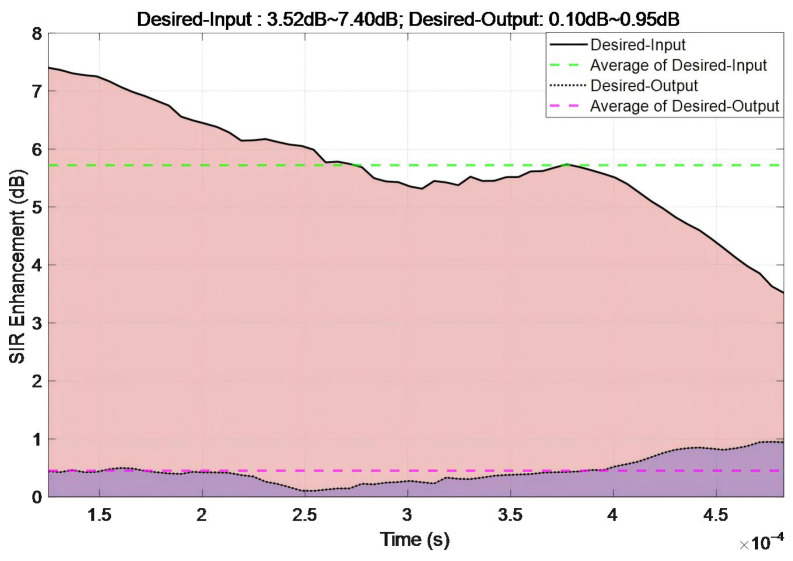
Temporal evolution of SNR enhancement under frequency-swept interference.

Figure 20 presents the temporal evolution of SIR enhancement under frequency-swept interference. The Desired-Input SIR ranged from 3.52 to 7.40 dB (mean: 5.72 dB). The Desired-Output SIR varied between 0.10 and 0.95 dB (mean: 0.45 dB). The proposed algorithm induced an SIR reduction (output–input) of 2.58–6.96 dB across measured points, with a mean reduction of 5.27 dB. These results confirm the algorithm’s efficacy in suppressing frequency-swept interference.

## 5. Conclusions

This paper introduces a novel VSS-LMS algorithm based on a modified versoria function that resolves the CR-SSE trade-off through the systematic optimization of parameters (α, β, and γ), thereby enhancing inherent anti-jamming capabilities. Its dynamic step-size mechanism, which couples instantaneous error autocorrelation with a restructured versoria mapping, enables rapid convergence (~40 iterations) and the lowest steady-state MSE across 5–15 dB SNRs compared to benchmarks (I-Sigmoid, M-Arctan, etc.). The algorithm achieves exceptional robustness with 39.75 dB SNR gain at −40 dB AWGN, and >75% PSD suppression against single-tone/narrowband/ swept-frequency jammers. Crucially, the modified versoria function eliminates complex operations, ensuring real-time feasibility of sensor array signal reception systems. In summary, this algorithm provides a high-performance, practical VSS-LMS solution with synergistic advantages in CR, MSE, and robustness, offering significant potential for sensor array signal reception systems in challenging environments. Future work will conduct field validation testing utilizing actual received signals from the receiver to rigorously evaluate performance under operational conditions.

## Figures and Tables

**Table 1:** Specific steps of traditional LMS adaptive filter algorithm.

Step	Operation	Mathematical Expression	Implementation Note
a	Initialize parameters	Set filter order L , step size μ , initial weights W(0)	Typically W(0)=0
b	Compute filter output signal	$y(n)=W^{T}(n)X(n)$	Requires L multiply-accumulate operations
c	Calculate error signal	e(n)=d(n)−y(n)	Real-time subtraction operation
d	Update weights	w(n+1)=w(n)+2μe(n)X(n)	Dominates computational load (O(L))
e	Check convergence	Repeat steps b–d to minimize MSE until convergence	$\mathrm{Termination}\;\mathrm{criteria}:\;\mathrm{Max}\;\mathrm{iterations}\;N_{max}$ (e.g., 1000)

**Table 2:** Function expressions of different VSS-LMS algorithms and corresponding values.

Algorithm	VSS-LMS Function	Parameters
K-Versoria	$\mu \left(n\right)=\beta \times \mathrm{artanh}\left(\alpha \times {\left[e\left(n\right)\right]}^{\gamma }\right)$	α = 0.01
		β = 4
		γ = 0.1
I-Sigmoid	$\mu \left(n\right)=\beta \times \frac{1-e^{-\alpha |e\left(n\right)e\left(n-1\right){|}^{\gamma }}}{1+e^{-\alpha |e\left(n\right)e\left(n-1\right){|}^{\gamma }}}$	α=0.1
		β=0.1
		γ=3
SVS	$\mu \left(n\right)=\beta \times \left(\frac{1}{1+e^{-\gamma \left|e\left(n\right)\right|}}-0.5\right)\left(1+\frac{1}{1+e^{-\alpha \left(\left|e\left(n\right)-1.5\right|\right)}}\right)$	α=23
		β=1.5
		γ=0.08
IVIS	$\mu \left(n\right)=\beta \times \left[\frac{1}{4}-\frac{e^{-\alpha \times \left|e\left(n\right)\right|}}{{\left(1+e^{-\alpha \times \left|e\left(n\right)\right|}\right)}^{2}}\right]$	α=3
		β = 0.5
M-Arctan	$\mu \left(n\right)=\beta \times \arctan^{\gamma }(\alpha |e\left(n\right)e\left(n-1\right)|)$	α = 50
		β = 0.03
		γ = 3
Proposed Algorithm	$\mu \left(n\right)=\alpha \left(\frac{1}{1+\frac{1}{\beta E{\left[e\left(n\right)e\left(n-1\right)\right]}^{\gamma }}}\right)$	α = 1
		β = 0.1
		γ = 0.1

**Table 3:** Mathematical formulations and parameter distributions of jamming interference types.

Interference Type	Interference Mathematical Function	Parameter Distribution
Single-Tone	$J_{ST}=P_{st}\exp\left(j\left(2\pi f_{st}t+\varphi_{st}\right)\right)$	Jamming Power $P_{st}$ = 0.3
		Carrier Frequency $f_{st}$ = 1.5 MHZ
		Carrier Phase $\varphi_{st}\;\sim \;\left(0,\;2\pi \right)$
Narrow Band	$J_{NB}=P_{nb}\cdot \mathrm{BP}\left(n\left(t\right);f_{nb},B_{nb}\right)$ Ideal Band-Pass Filtering $\mathrm{BP}\left(\cdot \right)$	Jamming Power $P_{nb}$ = 0.5
		Center Frequency $f_{nb}$ = 1.2 MHZ
		Bandwidth $B_{nb}$ = 200 kHZ
		Gaussian White Noise $n\left(t\right)$–(0, 1)
Sweep	$J_{S}=P_{w}\exp\left(j\left(2\pi f_{0}t+\pi \frac{f_{1}-f_{0}}{T_{swp}}t^{2}+\varphi_{w}\right)\right)$	Jamming Power $P_{w}$ = 0.5
		Sweep Period $T_{swp}$ = 1
		Upper Frequency $f_{1}$ = 4 MHZ
		Lower Frequency $f_{0}$ = 3 MHZ
		Carrier Phase $\varphi_{w}\;\sim \;\left(0,2\pi \right)$

**Table 4:** PSD characteristics under narrow-band interference.

Signal	Max PSD (dBW/HZ)	Min PSD (dBW/HZ)	Average PSD (dBW/HZ)
Input	−61.68	−74.02	−65.40
Desired	−80.94	−96.75	−87.69
Output	−77.05	−85.49	−82.16

**Table 5:** PSD characteristics under frequency-swept interference.

Signal	Max PSD (dBW/HZ)	Min PSD (dBW/HZ)	Average PSD (dBW/HZ)
Input	−56.69	−91.6872	−61.6
Desired	−74.03	−119.7989	−84.14
Output	−74.31	−101.73	−84.8

## Data Availability

The raw data supporting the conclusions of this article will be made available by the authors on request.

## Abbreviations

The following abbreviations are used in this manuscript:

CR	Convergence rate
SSE	Steady state error
VSS	Variable step size
LMS	Least mean square
RLS	Recursive least squares
AP	Affine projection
SNR	Signal-to-noise ratio
AWGN	Additive white Gaussian noise
MSE	Mean square error
PSD	Power spectral density
TF	Time frequency
JSR	Jammer-to-signal ratio
SIR	Signal-to-interference ratio
